# Endometriosis: new insights and opportunities for relief of symptoms

**DOI:** 10.1093/biolre/ioaf164

**Published:** 2025-07-24

**Authors:** Philippa T K Saunders, Andrew W Horne

**Affiliations:** EXPPECT Edinburgh, Centre for Reproductive Health, Institute for Regeneration and Repair, The University of Edinburgh, Edinburgh EH16 4UU, UK; EXPPECT Edinburgh, Centre for Reproductive Health, Institute for Regeneration and Repair, The University of Edinburgh, Edinburgh EH16 4UU, UK

**Keywords:** endometrium, chronic pain, infertility, diagnostic delay, neuro-angiogenesis, inflammation, macrophage, hormonal therapy, co-morbid

## Abstract

Endometriosis is a chronic neuroinflammatory disorder believed to impact on the wellbeing of more than 190 million women and people assigned female at birth. The defining hallmark of endometriosis is the growth of endometrial-like tissue as “lesions” outside the uterus. Most lesions are found in the pelvis and referred to as peritoneal (superficial), ovarian (endometrioma) or deep depending on location. Patients often suffer from persistent pelvic pain which can be worse during menstruation as well as fatigue, gastro-intestinal and urinary symptoms and mood disorders that impact quality of life. It is estimated 30–50% of patients with endometriosis may have problems conceiving. Diagnostic delay is ~7–9 years after first symptoms. There are currently no reliable biomarker(s). Advances in imaging have improved diagnosis of ovarian and deep subtypes but definitive diagnosis may require invasive laparoscopic surgery. Standard treatment options include surgery as well as drugs that suppress ovarian hormones which have unwanted side effects. New approaches to symptom management have been informed by the reframing of endometriosis as a multisystem disease. Genetic studies have identified shared risk factors with inflammatory and other chronic pain conditions. Alterations in hormonal, metabolic, and inflammatory pathways in samples from endometriosis patients have opened-up new avenues for medical therapy, including drug repurposing. There is increased interest in non-medical and self-management strategies including nutrition. In this narrative review we discus recent research studies and ongoing clinical trials which are addressing the need for novel approaches to reduce the impact of symptoms on quality of life.

## Teaser

Endometriosis is a multisystem inflammatory disorder for which new approaches to symptom management are being developed based on genetics, metabolic changes and self-management strategies.

## Graphical Abstract

**Graphical Abstract:**
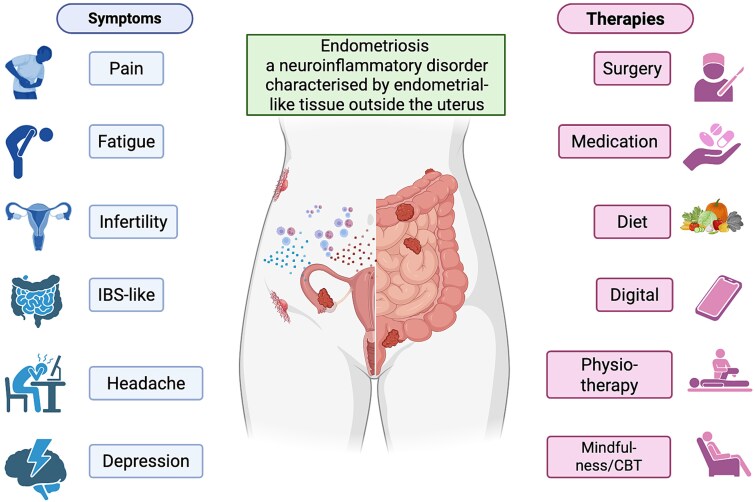

## Introduction

**Figure 1:**
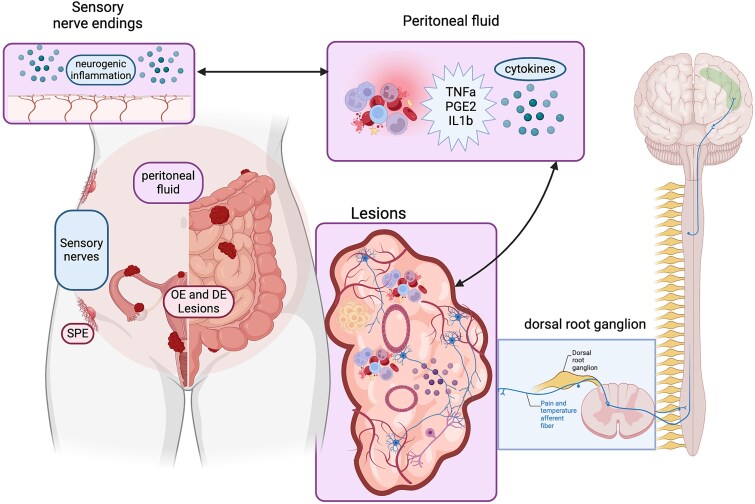
Pathophysiology of endometriosis. Endometriosis lesions are typically found within the pelvic cavity (individuals may have more than one subtype): superficial peritoneal (SPE), ovarian (OE) or deep (DE) subtypes. The presence of lesions is associated with a marked inflammatory response resulting in changes in numbers/types of immune cells present in peritoneal fluid and higher levels of cytokines, prostaglandins and growth factors (e.g., IL1beta, TNFalpha, PGE2) [35]. The peritoneum is innervated by somatic and visceral fibers, activation of which may lead to chronic pain, peripheral sensitization and increased visceral hyperalgesia [163]. Lesions contain stromal cells, areas of fibrosis, epithelial cells lining gland-like structures, and multiple subtypes of immune cells. Lesion survival is promoted by development of blood vessels (angiogenesis) and the associated growth of peripheral nerves (neuroangiogenesis) that link to the central nervous system [6, 35]. The combination of neoinnervation, peripheral sensitization and surgical intervention may all contribute to neuropathic mechanisms of endometriosis-associated pain. Endometriosis-associated pain involves both peripheral and central mechanisms including the somatosensory cortex, anterior insula, thalamus and brain stem with changes in the structure of and function of the brain similar to those in other chronic pain conditions [6]. Original figure was drawn using Bio render by PTKS and is adapted from one previously published in [116]: reproduced under a CCBY open access license.

Endometriosis is a chronic condition the hallmark of which is the presence of lesions (tissue resembling endometrium) in sites outside the uterus [1, 2] (Figure 1). Reports suggest that it may affect ~190 million women and those assigned female at birth [3]. This is likely to be an underestimate as some individuals may have lesions that remain undiagnosed [4] and access to medical facilities and experts able to make a definitive diagnosis may be limited in some countries. Endometriosis is associated with a wide range of debilitating symptoms, which can have a negative impact on well-being, day-to-day activities and life course potential [5]. Symptoms include chronic pelvic pain (often worse during menstruation), heavy menstrual bleeding, fatigue, urinary symptoms, abdominal bloating, and other gastrointestinal problems. Unsurprisingly, low mood, increased anxiety, and depression are also common [6, 7]. Notably, for many individuals the association of endometriosis with sub/infertility is also a considerable health burden with many women resorting to assisted reproductive technologies (ARTs). Once pregnant (either naturally or via ART) pregnancy complications including miscarriage and ectopic pregnancy are increased [8]. Placenta previa, hemorrhage and preterm birth are also higher in women with endometriosis [9].

## Subtypes of endometriosis and clinical staging

Endometriotic lesions contain endometrial-like stromal and epithelial cells, immune cells, new blood vessels and nerve fibers, and regions of fibrosis and hypoxia (Figure 1). Most patients have lesions, which are in the pelvic cavity and are referred to as three subtypes depending upon location—on the peritoneal wall so called superficial peritoneal (SPE), as cysts in the ovary (endometriomas/“chocolate cysts”, OE) and deep endometriosis (DE). Deep disease is characterized by nodules that penetrate below the surface of the tissue between the rectum and the vagina (known as recto-vaginal endometriosis), on the bowel or bladder and between the bladder and the uterus [10]. Notably, although rare compared to pelvic sites, endometriosis lesions can also occur in extra-pelvic sites including the thoracic cavity (diaphragm, lungs, trachea [11], in cesarean section scars [12] and around the sciatic nerve [13]).

As a complement to surgical diagnosis, several staging systems have been developed that focus on describing the location of lesions combined with extent of disease. A recent working group summarized these systems and their strengths and weaknesses [10]. The most widely used is still the revised scoring system of the American Society for Reproductive Medicine, used to determine the stage (ranging from I-IV, indicating “minimal” to “severe” endometriosis) on the basis of the type, location, appearance, and depth of invasion of the lesions [14]. Both the working group and the authors of several publications have highlighted the discordance between stage/lesion type as defined at time of surgery and severity of pain symptoms [15].

### Diagnosis

Achieving a definitive diagnosis following first reported symptoms is ~7 years on average although this can vary widely [16]. Reasons include lack of awareness among patients and primary care physicians, and the fact that patients with endometriosis present with symptoms that overlap with other conditions (e.g., IBS, bladder pain syndrome). Individuals subsequently diagnosed with endometriosis have a higher number of hospital visits compared to individuals without a diagnosis of endometriosis [17]. Although there have been fewer studies in adolescents, this group may present with atypical symptoms that can make endometriosis more difficult to diagnose. For example, a recent prospective study reported that adolescents (12–18 year olds) with endometriosis had earlier menarche, longer menstrual periods and more frequent headaches and nausea than those >18 years old [18]. This complements earlier work on the higher incidence of migraines in adolescents with endometriosis [19]. As many patients report having first symptoms before age 18 greater attention needs to be given to adolescents if we are to achieve reductions in delays to diagnosis.

### Surgery

While surgery (laparoscopy) is still considered the gold standard for diagnosis of endometriosis international guidelines now recommend that clinical examination and imaging (see below) should both be undertaken to provide patients with a “clinical” (or “working”) diagnosis of endometriosis to allow for early instigation of treatment [20]. In addition, the benefits of therapeutic laparoscopy for treating pain associated with SPE (the commonest endometriosis subtype) has been challenged [21] and a clinical trial is underway to evaluate whether removal of SPE improves patient’s pain symptoms or not [ESPRIT2; ISRCTN27244948]*.*

### Imaging

In center’s that are well equipped with specialist staff experienced in advanced pelvic ultrasound or MRI, these imaging modalities can be used as reliable/robust alternative to laparoscopy for diagnosis of OE or DE and they are recommended in some clinical guidelines [22]. Use of imaging in diagnosis should increase with new methods including application of artificial intelligence to improve analysis [23, 24]. More advanced imaging methods including positron emission tomography (PET) used in combination with CT (PET-CT) and radiotracers also show promise. These studies are benefitting from probes developed to detect cancer and fibrotic disease although studies with fluorodeoxyglucose highlight these may be complicated by uptake within the endometrium and ovaries of premenopausal women [25]. Another example are radiolabeled targets for proteins such as FAP (fibroblast activation protein-α) [26], which should target areas of fibrosis known to be present in lesions and might be beneficial in identifying the location SPE lesions and those outside the pelvis.

### Biomarkers

As surgery comes with risks and is expensive, and imaging technologies are not suitable for all subtypes of endometriosis, there have been many studies investigating putative biomarkers in body fluids including blood, saliva, and urine. While many have shown early promise, independent validation in large groups of individuals and commercialization has been challenging [27]. Recent studies have provided some promising results, with candidates including panels of miRNAs in serum and saliva [28, 29] claiming potential as diagnostics for SPE [30] and endometriosis-associated infertility [31]. Studies on the microbiome of patients with endometriosis are also increasing (see below) some of which are claiming fecal metabolites, such as 4-hydroxyindole might act as stool-based diagnostic biomarkers although further validation studies are required [32].

### Symptoms

Endometriosis patients may suffer from a complex mixture of symptoms and the reframing of endometriosis as a multisystem, neuroinflammatory condition has highlighted the importance of symptoms not being considered in isolation [33]. Iron deficiency is also higher in patients with endometriosis, and this may exacerbate symptoms, such as fatigue [34].

### Pain

Pain is a complex sensory experience that may be acute or chronic (months/years). It has been estimated that 80% of patients with endometriosis suffer from chronic pelvic pain [35]. Patients with endometriosis report excess pain during activities including sex, defecation, menstruation and urination (see Figure 1 in [6]) as well as unpredictable pain flares against a background of chronic pain [36].

Aberrant inflammation with changes in the phenotype of immune cells, increased biosynthesis of bioactive molecules including cytokines and prostaglandins, as well as “neuro-inflammation” (cross-talk between immune and nerve cells) are all considered as playing a key role in activation of both central and peripheral pain pathways in endometriosis [6]. Examples of the association between inflammation and increased pain include studies on immune cells in the human peritoneum [37], changes in ratios of neutrophils to lymphocytes in blood [38] and studies in mouse models of endometriosis all highlighting a potential role for innate immune cells in lesion growth and pain mechanisms [39, 40]. In a recent comprehensive review, Coxon and colleagues summarized the latest data from many studies on endometriosis-associated pain including evidence for involvement of both nociception and neuropathic mechanisms [41]. They also highlighted the evidence for an additional mechanism—nociplastic pain—a type of pain defined as one that reflects changes in how the nervous system processes pain signals [42]. Nociplastic pain is associated with symptoms including fatigue and sleep disturbances that are commonly experienced by patients with endometriosis [43].

Women with endometriosis have a higher incidence of other chronic pain/fatigue conditions including fibromyalgia and chronic fatigue syndrome [44]. Several studies have identified mechanisms related to cross-sensitization associated with comorbid pain conditions, such as bladder pain syndrome, irritable bowel syndrome (IBS), abdomino-pelvic myalgia, and vulvodynia [45], which have implications for patient-centered care.

### Infertility and pregnancy complications

Endometriosis lesions can be found in asymptomatic women and are detected in up to 50% of women seeking treatment for infertility, [46]. In a meta-analysis of papers published between 2018 and 2022, Vercellini and colleagues [47] summarized evidence of reduced pregnancy and live birth rates and an increased miscarriage rate in women with endometriosis. Whereas women with SPE or OE were not at increased risk of third trimester and neonatal complications, those with DE were at several-fold increased risk of placenta previa [47]. In a Scottish study based on hospital records women with all subtypes of endometriosis were also at found to be higher risk of ectopic pregnancy and preterm birth [9].

Studies on the mechanisms responsible for endometriosis-associated infertility have explored changes in the eutopic endometrium and reductions in ovarian reserve in those with OE with most data coming from individuals seeking fertility treatments including IVF [48, 49]. Age is a key determinant of success in IVF and, whilst pregnancy complications may still occur, success rates for patients with endometriosis appear comparable to those without lesions [50]. There is an ongoing debate as to whether surgery for endometriosis is beneficial, or not, when the patient’s priority is to achieve a pregnancy although this may depend on severity of disease and be contraindicated for OE [51].

### Gastrointestinal symptoms

Symptoms involving disturbances of the gastro-intestinal (GI) tract are often experienced by patients with endometriosis and their unpredictability can make them both hard to manage and lead to misdiagnosis. Those most often reported are like those present in patients with IBS including diarrhea, constipation, and flatulence. One large Danish study found women with endometriosis had increased risk of inflammatory bowel disease, Crohn disease, and ulcerative colitis [52]. A symptom often reported as causing distress to endometriosis patients is abdominal bloating (sometimes referred to as “Endo belly”) that tends to occur during the second half of the menstrual cycle [53]. Mechanisms for abdominal bloating are not fully understood but appear linked to an interplay between hormonal changes and gut inflammation with further work required to mitigate their impact.

A study on nearly 200 000 women found evidence for genetic links between endometriosis risk factors and those associated with GI disorders. For example, endometriosis patients were twice as likely to have a diagnosis of IBS and 1.4x more likely to have GORD (gastro-esophageal reflux disease) [54]. Notably, the authors of this paper postulated that the identification of shared risk loci could accelerate identification of therapeutic drug targets and drugs that could be repurposed to treat GI symptoms in endometriosis patients.

### Depression/anxiety

Patients with endometriosis report higher levels of negative mood, depression, and anxiety. Some investigations have linked depression to pain symptoms with a variety of mechanisms proposed including oxidative stress and gut dysbiosis [55]. Recent studies have provided new insights into genetic associations between endometriosis, anxiety and depression [56, 57]. For example, a study using data from UK Biobank, which included 8200 patients with endometriosis and 194 000 healthy controls, found that a diagnosis of endometriosis significantly increased the odds of having psychiatric disorders even when they accounted for potential confounders such as chronic pain, socioeconomic status, age, body mass index, and co-morbid conditions [57]. As part of this study a genome-wide association studies (GWAS) identified one locus, DGKB rs12666606, with evidence of pleiotropy between endometriosis and depression highlighting the need for research into shared risk factors.

In an online survey completed by 653 participants on their psychological well-being found self-compassion emerged as an important protective factor [58]. Complementing this survey is a study reporting results from 301 women with endometriosis that investigated whether negative perceptions of bodily external and internal stimuli (interoception) may also play a role in negative mood. They found pain severity significantly predicted depressive symptoms and their findings, and those of the survey [59], both support wider introduction of interventions to improve psychological well-being in personalized patient care of women with endometriosis particularly those among the young and recently diagnosed.

## Current strategies for medical and surgical management of symptoms

Standard treatment options for endometriosis-associated symptoms include analgesics, surgery and/or medical therapies that target hormone production/action, inflammatory processes, and pain pathways [6] [7]. Guidelines with recommendations for the use of these treatments have been published by national healthcare systems in several countries including the UK [https://www.nice.org.uk/guidance/ng73/chapter/recommendations], Canada [22] and Australia [https://ranzcog.edu.au/wp-content/uploads/Endometriosis-Clinical-Practice-Guideline.pdf] and by European Society of Human Reproduction and Embryology [20]. Management of patients wishing to get pregnant may also involve assessment of ovarian reserve and ART with a recent papers showing the impact (positive or negative) on surgical removal of lesions prior to ART [60, 61].

Long term use of some treatments (e.g., non-steroidal anti-inflammatories) is not recommended and the probability of recurrence of endometriosis-related pain, following surgical treatment is estimated at 40–50% by 5 years [62]. Medical therapies that suppress sex steroid production/action may also have unwanted side effects including blocking fertility or induction of menopause-like symptoms leading to high levels of dissatisfaction among patients. For all these reasons, there is an unmet need for a more personalized approach to symptom management which has stimulated studies and trials for new non-hormonal medical therapies as well as an increased adoption of non-medical approaches several of which are discussed below.

### New advances in our understanding of mechanisms that can contribute to the complex pathophysiology and etiology of endometriosis

In the last 100 years much of the research effort has focused on key research questions such as “Why does endometriosis affect some, but not all women?” and “What are the mechanisms that underpin the development of symptoms of varying severity?” In the opinion of some commentators, there has been too much emphasis placed on the “retrograde menstruation” theory of endometriosis [63] with the suggestion new theories need to be developed and tested [64]. In the last 5 years we, and others, have reviewed the many dozens of studies on patient samples and in animal models that have expanded our understanding of potential mechanisms contributing to pathophysiology [3, 6, 33, 65–68]. In the following sections, we highlight recent studies on this topic.

### Genetic studies have identified variants that increase risk of endometriosis and highlighted links to co-morbidities

Historical twin studies have reported the heritable component of endometriosis as ~50% [69]. The adoption of harmonized methods of recording patient data using EPHect guidelines [70] combined with methods to identity genetic variants using GWAS, Mendelian randomization and data from health care records has led to new insights into etiology [71–73]. Specifically, as with other complex diseases, individual genetic variants in the DNA sequence increasing endometriosis risk will have small effects so it is important to look for patterns in the processes that they may regulate [74]. In many GWAS, the most significant loci are associated with disease classified as stage III/IV, including OE, DE. Many studies also suffer from an over representation of samples from individuals with European ancestry [75, 76]. To date key pathways that have been identified by GWAS include Wnt signaling, genes implicated in hormonal regulation (estrogen, FSH) [71, 77], inflammation/immune cells [78] and coagulation factors [79].

Some of the most powerful data has been generated by comparison between data for endometriosis-associated loci and those for co-morbid conditions [72]. For example, Rahmioglu et al. [80] analyzed 60 674 endometriosis cases and 701 926 controls of European and East Asian descent and identified 42 significant disease associated loci. Notably they extended their study and found genetic correlations between endometriosis and 11 other pain conditions, including migraine, back and multisite chronic pain (MCP), and inflammatory conditions, including asthma and osteoarthritis [80]. Asthma was also found to be associated with risk factors implicated in sex hormone and thyroid signaling pathways [81]. Associations between endometriosis risk factors and those for depression [56], ovarian cancer [82], GI conditions [54], and reproductive disorders such as fibroids and endometrial cancer [74] have also been identified highlighting new avenues for research.

### Studies on the eutopic endometrium of patients with endometriosis have identified changes in gene expression and its regulation

The emphasis on retrograde menstruation as a key mechanism by which endometriosis lesions form in the pelvis has stimulated many studies. Evidence that changes in the endometrium within the uterus (“eutopic”) may contribute to formation, survival and progression of endometriosis lesions and development of symptoms that have already been the subject of expert reviews [3, 6, 68, 77, 83]. Some mechanisms identified include altered responses to normal steroid signaling that may partially explain the association between endometriosis and infertility [84], changes in microRNAs [85], shedding of stem/progenitors [68] and altered methylation and transcription of genes involved in tissue regulation [86, 87]. Studies on human samples have been complemented by those in animal models of endometriosis that have been used to validate changes in endometrium that may be therapeutic targets including macrophage subpopulations [88] the endocannabinoid system [89] and genes associated with progesterone signaling [90, 91]. Notably commentators have argued there is an over reliance of studies using cells and tissues derived from endometrium and that it is important the unique cellular and molecular profiles of endometrium and endometriosis lesions are both represented in preclinical models [92] as discussed below.

### Studies using patient samples have identified changes unique to lesions and the peritoneal microenvironment that have been used as the evidence for clinical trials

There have been a large number of studies using patient derived tissues/cells investigating whether cells/processes in endometriosis lesions have a phenotype, which is different to that of the eutopic endometrium (Figure 1). The aim of these studies is not only to gain a fundamental understanding of the lesion microenvironment and how/why the presence of lesions is associated with the diverse symptoms associated with endometriosis but also with the hope that lesions can be targeted by therapies which are disease modifying [6]. Complementing studies on lesions have been those comparing peritoneal fluid and the peritoneal wall of endometriosis patients and controls [2]. Highlights from a large body of work by many investigators have been the discovery that lesions develop new blood vessels and associated nerves which link the tissue to pain and other neural pathways (neuro-angiogenesis [93]), and a unique steroid microenvironment associated with altered expression of enzymes involved in metabolism of steroids [94, 95] and prostaglandins [96]. Additional insights have been gained from the application of single cell RNA technologies to evaluation of eutopic endometrium and endometriosis lesions [97]. Sarsenova et al [98] detected nine major cell types and evidence of altered co-activation of glycolytic and oxidative metabolism in perivascular and stromal cells of lesions, which may contribute to lesion growth.

These studies have also extended our understanding of the role(s) played by specific subtypes of immune cells and inflammatory mediators in endometriosis complementing evidence of supporting a role for macrophages in disease etiology [97, 99, 100] and pain mechanisms [37]. Comparison between peritoneal tissue samples (lesions, peritoneal wall from controls and endometriosis, mesothelial cells) identified alterations in the metabolic response of mesothelial cells in endometriosis patients [101] and increased expression of genes implicated in pain signaling including ion channels (TPV1, TRPA1, P3RX3, [102] and growth factors) [39]. Some of these findings have informed the design of clinical trials (Table 1).

**Table 1:** Examples of clinical trials targeting candidate pathways that are altered in endometriosis patients (clinicaltrials.gov).

Trial number	Title	Treatment	Primary outcome	Secondary outcome
NCT05101317 (China, USA, Poland)	A Study to Assess the Efficacy and Safety of HMI-115 in Subjects with Endometriosis-Associated Pain [monoclonal prolactin receptor agonist]	HMI-115 (60, 120 or 240 mg) s.c injection every 2 weeks 12 weeks	Change of dysmenorrhea (DYS) measured by Numeric Rating Scale (NRS) from Baseline to Week 12	Changes in non-menstrual pelvic pain, pain impact, bleeding heaviness, medication at baseline, 12 and 24 weeks
NCT02542410 (USA) [154]	Dopamine Receptor Agonist Therapy for Pain Relief in Women with Endometriosis: A Pilot Study	2 groups: Active comparator Norethindrone acetate 5 mg po daily x 6 months. Cabergoline 0.5 mg PO twice weekly x 6 months	Change in Score in Worst Pain Over the Last Month	Changes in Pain Interference Scores
NCT01190475 (USA, RCT)	BGS649 (aromatase inhibitor) Monotherapy in Moderate to Severe Endometriosis Patients	3 arms: placebo, low dose or high dose	Proportion of patient with 2 or more larger ovarian follicles (8 months)	Pharmokinetic profile of BGS649 (8 h)
NCT03654326 (USA) [109]	A Study to Evaluate the Efficacy and Safety of Gefapixant (MK-7264: P2X3 antagonist) in Women With Endometriosis-Related Pain	A gefapixant 45 mg tablet twice a day for approximately 8 weeks (2 menstrual cycles). Naproxen sodium 275 mg tablets will also be provided to participants for use as rescue medication for endometriosis-related pain.	Change in average daily pain in cycle 2, Percentage of AE, Percentage who discontinue	Change from baseline non-cyclic pain
NCT03840993 (USA)	Safety and Efficacy Study of MT-2990 (IL33 inhibitor) in Women With Endometriosis	16 weeks MT-2990 or placebo	Mean Change From Baseline to Week 16 in Non-menstrual Pelvic Pain Using a Pain Scale Ranges From 0 (None) to 3 (Severe)	Mean change other pain measures, analgesic use, self-image, social support dimension
NCT03373422 [terminated] [108]	A Study to Test Whether Study Drug BAY1128688 (AKR1C3 inhibitor) Brings Pain Relief to Women With Endometriosis and if so to Get a First Idea Which Dose(s) Work Best (AKRENDO1)	6 groups, 5 doses of BAY112688 and 1 placebo (12 weeks)	Absolute change in mean pain of the 7 days with worst EAPP comparing the 28-day baseline cycle (baseline period of daily pain recordings) to the 28-day end of treatment cycle (daily pain recordings during the last 28 days of the treatment period)	Incidence of treatment adverse events
NCT05560646	A Study to Investigate Efficacy and Safety of OG-6219 (17betaHSD1 inhibitor) in 3 Dose Levels Compared With Placebo in Participants Aged 18 to 49 With Moderate to Severe Endometriosis-related Pain (ELENA)	4 groups; 3 doses OG6219 and one placebo group 16 weeks	Change in mean endometriosis-related pelvic pain score between 1^st^ and last treatment cycle. Safety/tolerability	Changes in non-cyclical pain, EHP30, vaginal bleeding. ECG. Plasma levels/cmax

### New and emerging medical therapies

Our review of registered clinical trials testing medical therapies in women with endometriosis reveals many that are focused on improved formulations of hormonal therapies. Examples include oral formulations of GnRH antagonists such as merigolix, elagolix, linzagolix, and relugolix, with the latter having significant efficacy in reducing heavy menstrual bleeding in women with fibroids and pelvic pain in women with endometriosis [103]. Relugolix has recently being approved for endometriosis patients in the UK and under the trade name Ryeqo it has been authorized by the European Medicines Agency.

Whilst effective against pain in many women these new formulations may still cause unwanted side effects similar to those of menopause and are not suitable for women wishing to become pregnant leading to increasing interest in non-hormonal therapies.

Two major avenues are being explored by clinical trials—the first includes targets identified from studies on patient samples (Table 1, and discussion above) and the second drugs that have already been effective in mitigating symptoms in co-morbid conditions, such as IBS and migraine [104]. Examples of the former include targets that are over-expressed in endometriosis lesions such as the enzymes AKR1C3 and mPGES1, which are involved in biosynthesis of prostaglandins implicated in inflammatory pain pathways [105, 106]. Highly potent antagonists of mPGES1 are being tested in clinical trials [107] although the outcome is not yet known. Unfortunately several trials have been disappointing. For example, the phase IIa trial of an AKR1C3 inhibitor had to be terminated early due to hepatoxicity [108] and a P2X3 receptor antagonist had a high rate of taste-related adverse events and high dropout leading to inconclusive results (Table 1, [109]). Trials targeting inflammatory chemokines such as interleukin 8 (IL8) that are higher in the peritoneal fluid of women with endometriosis [110] appear more promising. In recent work, Nishimoto–Kakiuchi and colleagues developed a novel long-acting recycling antibody against IL-8 (AMY109), which they have tested both in a monkey model and in a phase 1 clinical trial with promising results [111]. A multi-center phase 2 trial is currently underway [ISRCTN15654320, ACERS clinical trial] with results expected in 2026.

In our own Center we have focused on drug repurposing as a way of accelerating new therapies for endometriosis-associated symptoms. One example is the enzyme inhibitor dichloroacetate (DCA), which is already approved for use to treat some metabolic disorders in children. We have shown that the peritoneal fluid of women with endometriosis has higher concentrations of lactate and that one source of this lactate are the mesothelial cells lining the cavity which have an altered metabolic profile [101]. When DCA was tested in our model systems, it was able to correct this phenotype [112] leading to DCA being tested in a small open label clinical trial [113] with a bigger RCT due to start soon. Other opportunities for drug repurposing may be informed by the genetic studies discussed above that have identified shared risk factors for conditions with shared symptoms such as pain (migraine) and GI disturbance (IBS) [54, 104]. Examples include Calcitonin gene-related peptide (CGRP) antagonists (Gepants) and CGRP monoclonal antibodies for prevention and treatment of migraine [114] both of which are promising given reports that CGRP signaling is involved in endometriosis-associated pain [115]. In a study on links between endometriosis and GI conditions, Yang et al identified some candidates genes for drug repurposing [54] highlighting, *CCKBR*, that encoded a protein targeted by two drugs Proglumide and Netzepide not yet tested in endometriosis patients and *PDE4B* the product of which is targeted by Pentoxifylline, already being tested for endometriosis (phase III) and IBS (phase IV) in separate trials.

**Figure 2:**
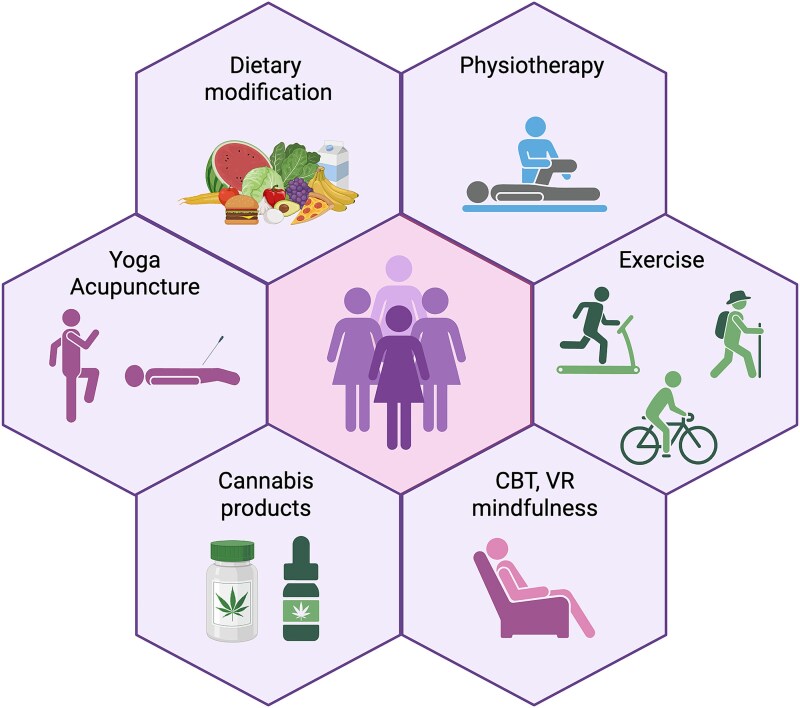
Therapies used to alleviate symptoms including pain, depression, and fatigue associated with endometriosis for which there is evidence of positive benefit. Original figure drawn using Bio render by PTKS.

In addition to approaches using pharmaceutical grade drugs, there is an increasing use of compounds derived from natural sources including cannabis products [116] (Figure 2). Patients report use of cannabis-derived products has allowed them to reduce their use of pain medications and to experience improved sleep [117, 118] and that they would recommend it to a friend or relative with endometriosis [119]. One challenge for adoption of cannabis-based products into routine health care is the lack of evidence from RCTs using formulations that contain known concentrations of cannabidiol, and minimal amounts of psychoactive compounds such as tetrahydrocannabinol, as the latter is associated with side effects including sleepiness and impaired cognitive function [116].

### Non-medical approaches to management of symptoms

There is increasing evidence that use of non-medical therapies including physiotherapy, exercise, diet [55, 120, 121], or devices such as VR headsets [122, 123] may be beneficial to patients (Figure 2) and several of these are also being evaluated in clinical trials (Table 2). Some of the evidence to support non-medical approaches are summarized below.

**Table 2:** Examples of clinical trials investigating lifestyle factors or dietary modification for management of endometriosis symptoms (clinicaltrials.gov).

Trial number	Title	Intervention	Primary outcome	Secondary outcome
NCT05175248 (USA)	Nutritional Intervention for Endometriosis	Intervention group participants will adopt a low-fat, plant-based diet for 12 weeks. Controls normal diet. Both groups vitamin supplement	EHP-30 score, Biberoglu and Behrman Scale: change from baseline, Blood tests for biomarkers of inflammation (hsCRP, TNF-alpha, IL-1 beta and IL-6).	Microbiome, body weight, lipids, oestrogens, brain-derived neurotrophic factor (BDNF) and IL-10
NCT05433909 (Italy)	Microbiota and Immunoassay in Women With and Without Endometriosis: a Pilot Study	Diagnostic Test: Blood, fecal, vaginal and endometrial liquid samples. Before and after surgery	Intestinal, vaginal and endometrial microbiota in patients with and without endometriosis before and after surgery	molecular and immunological characteristics of the inflammatory environment of endometriotic lesions and peripheral blood changed from women with and without endometriosis
NCT05983224 (Iran)	Effect of Quercetin Supplementation on Endometriosis Outcomes	The intervention group will receive two 500 mg quercetin tablets daily, after breakfast and lunch: for 12 weeks. Controls placebo	Serum TNFa, IL6	Serum testosterone, E2, IGF1, LH, FSH, P4
NCT04259788 (USA)	An Alternative Healthy Eating Index (AHEI) Dietary Intervention to Reduce Pain in Women With Endometriosis	The intervention group will receive in-person dietary counseling from a registered dietitian to help participants consume a diet that is consistent the AHEI dietary guidelines. 12 weeks. Controls no dietary intervention	Pain measurement VAS pain score, EPHect participant questionnaire using the pain catastrophizing scale, physical and mental components of QoL questionnaire, measurement IL6, IL1b, C reactive protein, TNFa, TNFR2	Measurements at baseline, 4, 8 and 12 weeks (no secondary)
NCT06660043 (France)	Influence of Bodily Practices on the Quality of Life of Women With Endometriosis (PCKendo)	3 arms: 40 min circuit training, physical therapy with massage and Transcutaneous Electrical Nerve Stimulation in a 40 min session, 25 min circuit training session and 15 min physical therapy	EHP30 questionnaire at 6 months	
NCT06332560 (The Netherlands)	Pain in Endometriosis And the Relation to Lifestyle (PEARL)	Anti-inflammatory diet based on the Dutch Dietary Guidelines or CBT or a combination of both interventions for 12 weeks	Change in pain intensity (NRS scale), change in inflammatory characteristics of menstrual effluent	Change in EHP30, QoL assessment, hair scalp cortisol, vaginal/gut microbiome, adherence to diet (3 mo)
NCT05831735 (France)	The CRESCENDO Program (inCRease Physical Exercise and Sport to Combat ENDO) (CRESCENDO)	3 arms. 6 months. Control—video only: Physical activity (video, 1-3 h physical activity viva videoconference; Physical activity plus 6 sessions of education/discussion groups	Change in perceived pain and fatigue, EHP30, change in physical activity between start and 6 months	Change in self-image, social support, motivation

### Dietary modification including supplements

Surveys of patients, as well as extensive coverage on social media, have highlighted the use of specific diets and supplements as strategies to manage symptoms such as pain, bloating and those affecting the GI tract [55, 124–126]. Diet, including the use of probiotics, vitamins, supplements, and drinks such as alcohol and coffee, may all impact on the gut [127], which has a diverse population of microorganisms (the “microbiome”) increasingly acknowledged as playing a major role in health [128, 129]. In addition to the well-known impact of the microbiome on gastrointestinal symptoms, there is a large body of work that has identified the gut-microbiota-brain axis that communicates closely with the immune system as having an impact on neuronal pathways including those involved in pain signaling [128]. In a 2012 study, 207 endometriosis patients followed a gluten-free diet for 12 months, after which 75% reported an improvement of pain symptoms and all participants experienced an increase in physical and mental health [130]. A study of 160 women with IBS, 36% of whom had endometriosis, used a 4-week dietary intervention with the low-FODMAP diet: 72% of those with endometriosis reported an improvement in bowel symptoms, compared to 49% of those with IBS alone [131]. In a recent single center pilot study, 62 participants who were encouraged to follow the low FODMAP diet (*n* = 22) or an “endometriosis” diet (*n* = 21) for 6 months reported they had improved bloating, less pain and better QoL [132]. These data have encouraged larger studies including prospective trials (Table 2) and have been complemented by studies exploring differences in the gut, oral or vaginal microbiome in women with endometriosis as a diagnostic target [133].

Alternatives to diet include use of supplements, such as magnesium, for which there is some data on effectiveness of intravenous administration [134] and quercetin which is being evaluated in a RCT (Table 2). A wide variety of traditional Chinese medicines are already on the market some of which have been tested in clinical trials showing potential benefits [135] although larger better controlled RCT are still required to inform best practice [136].

### Physical therapies and exercise

Pelvic floor physical therapy that targets the muscles and tissues of the pelvic floor can help alleviate pelvic pain caused by endometriosis and is popular with patients particularly those experiencing muscle spasms [120]. The impact of physical therapies has been assessed a few RCT. For example, “Physio-EndEA” consisted of a 1-week lumbopelvic stabilization learning phase followed by an 8-week phase of stretching, aerobic, and resistance exercises focused on the lumbopelvic area supervised by a physiotherapist [137]. When the program was applied to 16 women, there was high rates of satisfaction and adherence with positive effects on pain/sensitization and QoL [138]. In a RCT of women with DE (17 intervention, 13 control) who had five individual sessions of pelvic floor physiotherapy the only positive impact was a tendency to reduced constipation [139]. In another RCT, pelvic floor muscle training was undertaken daily and exercise also assessed using a questionnaire: to date results have focused on experiences of those participating which have been generally positive [140]. Recruitment to the CRESCENDO Program (Table 2) is currently underway and results of this trial will be valuable in developing guidance for physical exercise programs.

### Yoga and acupuncture

Yoga has been widely tested as a strategy to reduce the impact of pain on quality of life with positive results reported for chronic primary pain and methods including remote delivery [141, 142]. Some RCT have also been conducted to evaluate its effectiveness for women with endometriosis. In a study by Goncalves and colleagues, 40 women were randomized to participate (or not) in an 8 week yoga intervention [143]. The yoga practice resulted in a reduction in levels of chronic pelvic pain and an improvement in QoL. In a more recent study involving 42 women who followed a program consisting of 8 weeks of conventional therapy followed by 8 weeks of 90 min yoga, the yoga had a positive impact on pain and QoL and the authors suggested it should be recommended to women with endometriosis [144].

There are several different types of acupuncture, which have been used to relieve symptoms associated with endometriosis including chronic pelvic pain [145]. In a comprehensive review of 23 RCTs of different methods delivering acupuncture-related therapies the authors explored the risk of bias concluding that there was good evidence for a positive impact on symptoms but that bigger trials are needed [146].

### Mindfulness, cognitive behavior therapy and virtual reality

Mindfulness is a complex psychological concept that has been identified in numerous studies as a protective factor against stress linked to resilience and coping mechanisms [147]. Notably mindfulness training and psychological support delivered via apps, or in person, is now being tested as a strategy to help women overcome the experience of living with endometriosis particularly its impact on negative thoughts and feelings [148, 149]. To date, results from small scale trials have been mixed with some reporting improved QoL without improvement in pain [150] whereas others have recorded improvements in both pain and psychological stress [151]. Building on these data a digital intervention called MY-ENDO (Mind Your ENDOmetriosis) is being tested to see if a mindfulness- and acceptance-based endometriosis self-management intervention can help women how to manage and reduce negative physical, psychological, and social consequences of endometriosis [152] with initial feedback being generally positive.

Cognitive behavior therapy (CBT) is a talking therapy designed to help individuals overcome negative thoughts and is often used to help manage symptoms of anxiety and depression. In a recent RCT of patients with the chronic inflammatory condition rheumatoid arthritis it was found to be similar to mindfulness-based stress reduction in reducing pain and depression [153]. In a RCT, the efficacy of CBT in alleviating depression, stress, pain perception, and improving the quality of life was assessed in 52 patients with endometriosis with a significant positive change recorded in all patients in the intervention group [154]. This complements studies which have shown patients believe CBT should be offered in conjunction with endometriosis surgery. This is currently being tested in a clinical trial of 100 women the results of which are yet to be reported [155, 156]. A preliminary case–control study reported reduced levels of depression, anxiety, and stress in patients receiving CBT sessions in additional to usual care in hospital [157].

Virtual reality (VR) is emerging as a promising tool for pain management by immersing individuals in an alternative environment and has shown to be successful in reducing postoperative pain [158]. VR can also be designed to incorporate CBT and mindfulness techniques. Endocare is a VR solution that has been evaluated in a RCT in women with pelvic pain due to endometriosis [122, 123]. The use of the VR was undertaken by patients in their own homes and the study included equal numbers on the test and sham control groups (*n* = 51 in each). Patients used the VR headsets twice daily for at least 2 days (max 5 days) starting on the first day of painful periods with pain perception measured before and 60, 120 and 180 min after each treatment. The mean perceived pain relief was significantly higher on days 1 and 2 and resulted in reduced pain medication. Together with some smaller studies [159] these results suggest VR should be explored as part of pain management strategies particularly in the post operative period or during pain flares.

#### Apps

In the last 10 years, we have seen an explosion in the development of phone-based apps by research groups and private enterprise (so called FEMtech industries), which have been widely used for tracking of menstrual symptoms and fertility [160]. More recently, some apps have been updated and adapted to track symptoms in individuals with endometriosis to gain data that can be combined with methods such as machine learning [161, 162]. One example is the Lucy App that is being used to collect self-reported information on symptoms of endometriosis, mental and physical health, nutritional, and other lifestyle factors from 5000 women with confirmed endometriosis, compare this to the same number without endometriosis that will be analyzed by machine learning [161]. Data from apps are being complemented by studies using personal devices such as smart watches and activity monitors for patient tracking [162]. As algorithms improve and the amount of data is increased it is hoped that objective measures such as those from smartwatches can be used in clinical trials to complement data from patient reported questionnaires and to empower patients with information to complement self-management strategies including diet and exercise (Figure 2).

## Summary and conclusions

In the last 20 years we have increased our understanding of the pathophysiology of endometriosis with less emphasis on retrograde menstruation as the only explanation for the formation of lesions [63], a greater awareness of inflammatory processes as a key driver of symptoms such as pain [163] and new insights from genetic studies that have identified shared risk factors with other pain and inflammatory conditions [80]. Endometriosis researchers remain concerned that slow progress is being made towards improvements in care due to the complexity of symptoms and poor understanding of how these are driven by pathophysiology [64]. There have been repeated calls for more research into the causes of this challenging disorder so that time to diagnosis can be reduced [164] but in common with other conditions considered specific to women’s reproductive function endometriosis remains underfunded.

Advocacy and patient awareness have promoted a more holistic approach to treatment and increased adoption of self-help strategies that are helping some individuals reduce the severity of symptoms and improve QoL [121]. With a wide range of options now emerging it is important that patients are given advice on how they might adopt self-help strategies such as the use of apps, adjustments to their diet, CBT/mindfulness training and physical therapies as a complement to any conventional medical/surgical treatment.

The reframing of endometriosis as multisystem disorder, which has diverse symptoms [33], has been a big step forward in driving new initiatives for non-invasive diagnosis and therapies, which have less side effects than those currently recommended. To realize the goal of a more personalized approach to care of endometriosis patients we need to ensure this is delivered by multidisciplinary teams that include dieticians, physiotherapists, experts in mental health support as well as clinicians providing surgical and medical interventions.
